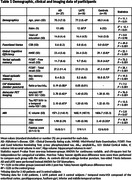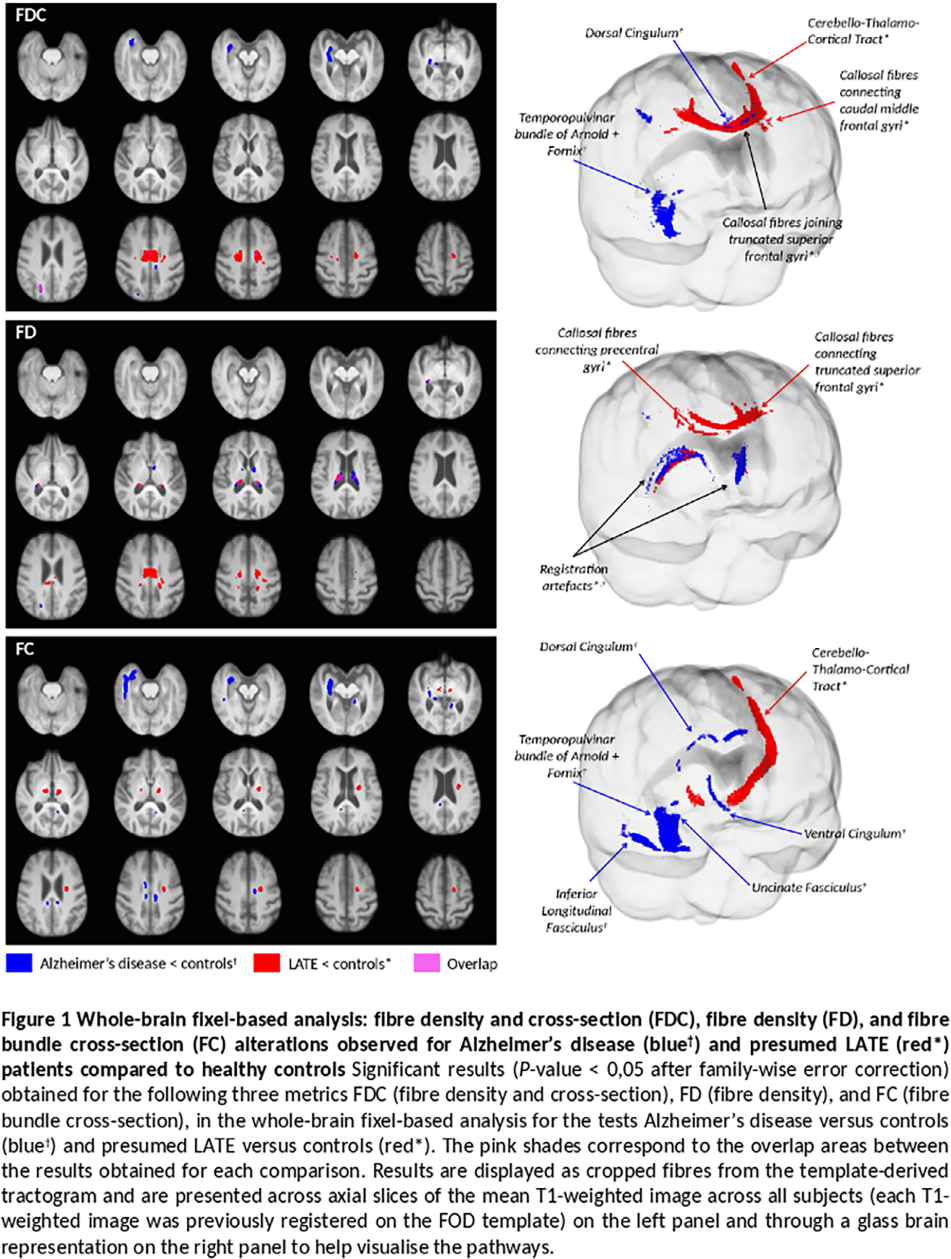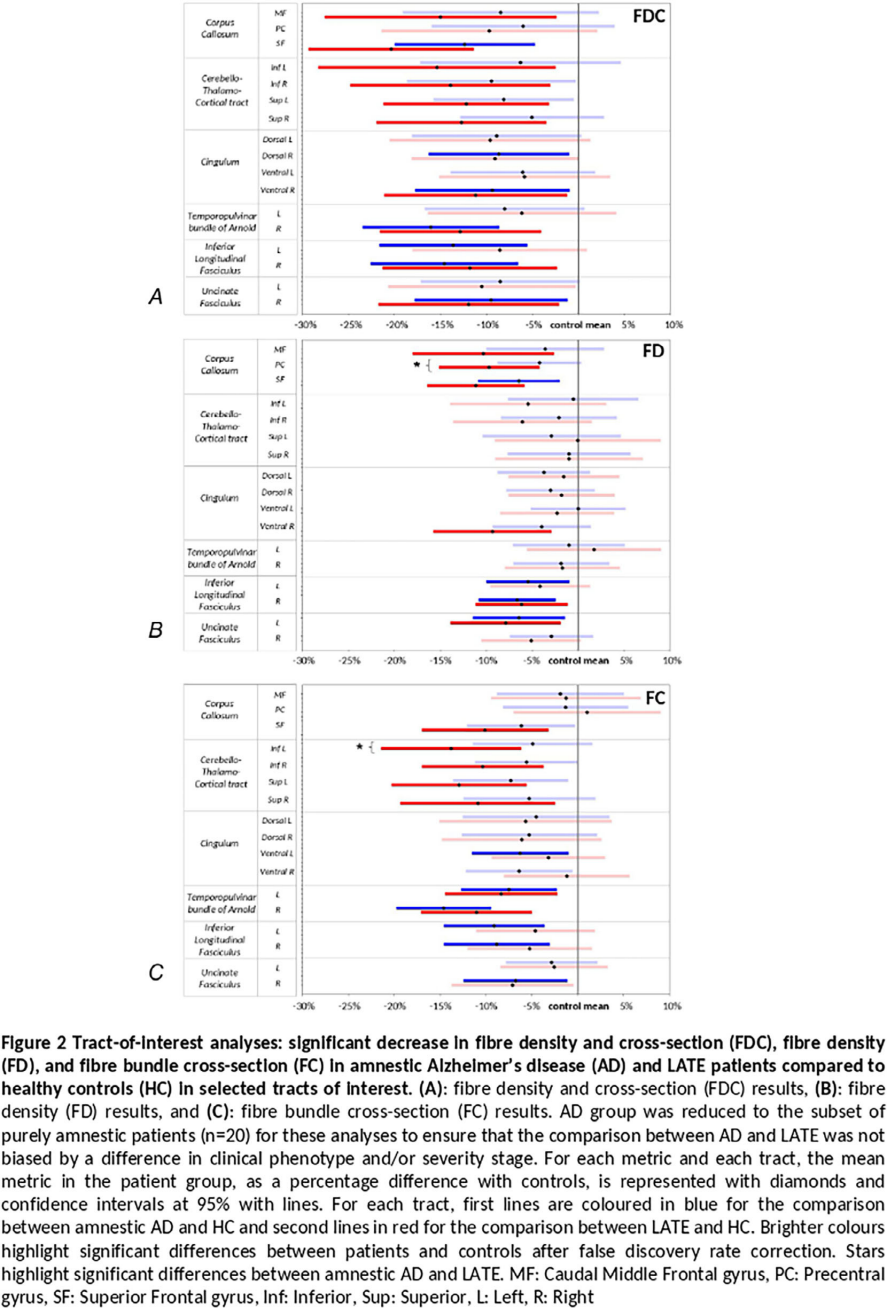# Investigating differences in white matter fibre bundle alterations between amnestic Alzheimer’s disease and LATE: A fixel‐based analysis

**DOI:** 10.1002/alz.088684

**Published:** 2025-01-09

**Authors:** Aurélie Lebrun, Julien Lagarde, Yann Leprince, Pauline Olivieri, Michel Bottlaender, Marie Sarazin

**Affiliations:** ^1^ UNIACT, NeuroSpin, CEA Paris‐Saclay, Université Paris‐Saclay, Gif‐sur‐Yvette France; ^2^ Université Paris‐Saclay, CEA, CNRS, Inserm, BioMaps, Orsay France; ^3^ Unit of Neurology of Memory and Language, Université Paris Cité, GHU Paris Psychiatry and Neurosciences, Hôpital Sainte Anne, Paris France; ^4^ Université Paris‐Saclay, UNIACT, Neurospin, Joliot Institute, CEA, Gif‐sur‐Yvette France; ^5^ Neurology of Memory and Language Department, GHU Paris Psychiatrie & Neurosciences, Hôpital Sainte‐Anne, Paris France

## Abstract

**Background:**

Typical Alzheimer’s disease (AD) and Limbic‐predominant Age‐related TDP‐43 Encephalopathy (LATE) are two neurodegenerative diseases that present with a similar initial amnestic clinical phenotype but have distinct proteinopathies. AD is characterised by ß‐amyloid plaques and intraneuronal neurofibrillary tangles, while LATE is characterised by abnormal neuronal TDP‐43 protein. With reference to the prion‐like hypothesis regarding the propagation of proteinopathies, investigating white matter fibre bundle alterations could provide new insights into the propagation pathways of specific proteinopathies. Because both AD and LATE have a similar initial limbic phenotype but are distinguished by their proteinopathies, they represent a good model to analyse whether white matter fibre bundle alterations differ according to the pathophysiology. We investigated these alterations in AD and presumed LATE using high quality diffusion MRI and fixel‐based analysis.

**Method:**

We included AD patients (*n*=27, CDR≤1), presumed LATE patients (*n*=18, CDR≤1), and controls HC (*n*=19) using strict pathophysiological criteria, including CSF biomarkers, amyloid and tau PET imaging, and the absence of clinical atypia after two years of follow‐up. Both AD (MMSE=24.5) and LATE (MMSE=24.4) exhibited equivalent episodic memory impairment and hippocampal volume. Using multi‐shell diffusion MRI, we first computed a whole‐brain fixel‐based analysis controlling for age, sex, MMSE, and intracranial volume to explore differences in FD (fibre density), FC (fibre‐bundle cross‐section) and FDC (fibre density and cross‐section) between groups, and to identify tracts of interest. We then reconstructed the identified tracts and performed tract‐based analyses with the same covariates to compare (i) AD versus HC, (ii) LATE versus HC, and (iii) AD versus LATE.

**Result:**

We found that both AD and LATE exhibited white matter alterations in tracts of the temporal and limbic lobes (ventral cingulum, inferior longitudinal fasciculus, uncinate fasciculus, temporopulvinar bundle of Arnold). In addition, LATE patients showed more spatially extended alterations in callosal fibres and in fibres of the cerebello‐thalamo‐cortical tract that end near the pre‐supplementary motor area.

**Conclusion:**

White matter fibre bundle alterations in AD and LATE exhibit differenciable patterns that are consistent with the staging system of tau or TDP‐43 accumulation. These findings also shed light on the temporopulvinar bundle of Arnold, that we found significantly altered in both diseases.